# Childhood dietary patterns and cardiovascular risk factors in adolescence: results from the Avon Longitudinal Study of Parents and Children (ALSPAC) cohort

**DOI:** 10.1017/S1368980016001592

**Published:** 2016-06-24

**Authors:** Caroline J Bull, Kate Northstone

**Affiliations:** School of Social and Community Medicine, University of Bristol, Oakfield House, Oakfield Grove, Bristol BS8 2BN, UK

**Keywords:** Childhood, Adolescence, Dietary patterns, CVD, ALSPAC

## Abstract

**Objective:**

To investigate the prospective associations between dietary patterns in childhood and CVD risk in adolescence.

**Design:**

Prospective cohort study. Exposures were dietary patterns at age 7, 10 and 13 years derived by cluster analysis. Outcomes were physiological and biochemical cardiovascular risk markers.

**Setting:**

Avon Longitudinal Study of Parents and Children (ALSPAC), UK.

**Subjects:**

Children (*n* 2311, 44.1 % male) with complete data available.

**Results:**

After adjustment for known confounders, we observed an association between being in the ‘Processed’ and ‘Packed lunch’ dietary pattern clusters at age 7 and BMI at age 17. Compared with the ‘healthy’ cluster, the OR (95 % CI) for being in the top 10 % for BMI was 1·60 (1·01, 2·55; *P*=0·05) for the ‘Processed’ cluster and 1·96 (1·22, 3·13; *P*=0·005) for the ‘Packed lunch’ cluster. However, no association was observed between BMI and dietary patterns at age 10 and 13. Longitudinal analyses showed that being in either the ‘Processed’ or ‘Packed lunch’ cluster at age 7 was associated with increased risk of being in the top 10 % for BMI regardless of subsequent cluster membership. No associations between other cardiovascular risk measures and dietary patterns were robust to adjustment for confounders.

**Conclusions:**

We did not find any consistent evidence to support an association between dietary patterns in childhood and cardiovascular risk factors in adolescence, with the exception of BMI and dietary pattern at age 7 only. However, the importance of dietary intake in childhood upon health later in life requires further investigation and we would encourage the adoption of a healthy diet as early in life as possible.

Foods are generally consumed in combination; therefore dietary recommendations should consider the diet as a whole, as well as focusing on individual foods or nutrients. Dietary intake throughout the life course is involved in the development of lifestyle-associated diseases, such as CVD and obesity, both of which are major health concerns in the UK^(^ ^1^ ^)^. The literature is increasingly supportive of childhood origins for a number of chronic diseases. It has been found that atherosclerotic development is initiated in childhood, long before clinical presentation^(^ ^2^ ^,^ ^3^ ^)^, and studies have previously linked childhood obesity with CVD in adulthood^(^ ^4^ ^)^. However, some evidence is less supportive^(^ ^5^ ^)^ and a recent systematic review concluded that childhood obesity could not be used as an independent predictor for CVD risk^(^ ^6^ ^)^.

Regardless of any association between childhood phenotypes and adult disease, there is evidence to support that behaviours (e.g. dietary intake) established in childhood will be maintained into adolescence and adulthood^(^ ^7^ ^,^ ^8^ ^)^. As diet and obesity are inextricably linked, and the literature links obesity and CVD, food behaviours established in childhood/adolescence may ultimately go on to affect adult cardiovascular (CV) health.

Collinearity between the intakes of individual dietary factors means that dietary patterns can be used to assess their cumulative effects; examining a single food or nutrient is often inappropriate as the impacts can be too small to detect. Dietary patterns are derived primarily via three statistical methods: cluster analysis (CA), principal component analysis and reduced rank regression. CA and principal component analysis are both purely data driven (whereas reduced rank regression extracts patterns that are characterised by pre-specified nutrient intakes and are thus likely to be related to particular outcomes of interest^(^ ^9^ ^)^) and have been found to give similar descriptive patterns in the Avon Longitudinal Study of Parents and Children (ALSPAC) at 7 years of age^(^ ^10^ ^)^. CA groups individuals into mutually exclusive categories at each time point, whereas principal component analysis and reduced rank regression both score individuals for each pattern obtained. Four clusters have been observed in ALSPAC using CA on food diary data at 7, 10 and 13 years of age^(^ ^11^ ^)^. We have chosen to use CA in the present study in order to clearly differentiate between the patterns obtained.

The associations between dietary patterns at ages 7, 10 and 13 years and CV risk factors at age 17 years were investigated in ALSPAC cross-sectionally, using regression modelling. It was hypothesised that there would be an association between dietary patterns (‘Healthy’, ‘Processed’, ‘Packed lunch’ or ‘Traditional’) and measured subclinical CV risk factors (physiological: systolic blood pressure (SBP), diastolic blood pressure (DBP) and BMI; biochemical: LDL cholesterol (LDL-C), HDL cholesterol (HDL-C), total cholesterol (TC), TAG and glucose), such that a ‘healthy’ dietary pattern would infer decreased risk.

## Methods

### The study population: Avon Longitudinal Study of Parents and Children

ALSPAC is a longitudinal cohort study established in the early 1990s to investigate determinants of health and disease^(^ ^12^ ^)^. In summary, 14 541 eligible pregnant women were enrolled at baseline. From these pregnancies 13 988 children were alive at 12 months of age. Later phases of enrolment resulted in an additional 713 children being enrolled and are also included in the current analysis^(^ ^12^ ^)^. The study website contains details of all the data that are available through a fully searchable data dictionary (http://www.bris.ac.uk/alspac/researchers/data-access/data-dictionary/).

### Assessment of diet

Dietary data were collected at each time point using a standardised protocol. Children were invited to a hands-on clinic-based assessment at age 7 (mean age=7 years 7 months, sd=4 months), age 10 (mean age=10 years 8 months, sd=3 months) and age 13 (mean age= 13 years 10 months, sd=2 months). Three-day food diaries were sent to participants prior to each clinic visit. Parents/carers were asked to record the consumption of all foods and drinks over two weekdays and one weekend day at age 7 years. At ages 10 and 13 years participants were asked to record this information themselves (with help from parents if necessary). Interviews were conducted at each clinic visit by a nutritionist in order to clarify portion sizes and any discrepancies in the recorded information. Twenty-four-hour recalls were conducted if the child did not bring their diet diary with them (<10 % at each time point). Food diary data were processed using DIDO (Diet In, Data Out), a program developed by the MRC Human Nutrition Research Unit (Cambridge, UK), as described previously^(^ ^13^ ^)^. The present study uses dietary patterns previously derived using CA^(^ ^11^ ^)^. Briefly, this involved grouping the participants based on their consumption of sixty-two food groups using *k*-means cluster analysis. Four diet clusters were identified at each age with similar distribution across time. These clusters were labelled ‘Processed’, ‘Traditional’, ‘Packed lunch’ and ‘Healthy’. Individuals grouped in the ‘Healthy’ cluster consumed on average more non-white bread, reduced-fat milk, cheese, yoghurt and fromage frais, butter, breakfast cereal, rice, pasta, eggs, fish, vegetable and vegetarian dishes, soup, salad, legumes, fruit, crackers and crispbreads, high-energy-density sauces (e.g. mayonnaise), fruit juice and water. Those in the ‘Processed’ cluster had a higher mean consumption of processed meat, pies and pasties, coated and fried chicken and white fish, pizza, chips, baked beans and tinned pasta, chocolate, sweets, sugar, and diet and regular fizzy drinks compared with the other clusters. Those in the ‘Traditional’ cluster had a higher mean consumption of red meat, poultry, potatoes, vegetables, starch-based products, low-energy-density sauces, puddings, tea and coffee. Finally, the fourth cluster was labelled ‘Packed lunch’ and individuals grouped in this cluster had a higher mean consumption of white bread, margarine, ham and bacon, sweet spreads, salty flavourings, crisps, biscuits, diet squash, tea and coffee (school-aged children typically eat these foods in packed lunches).

### Assessment of cardiovascular risk

The outcome measures used in the present analysis were collected during a clinic visit conducted when participants were age 17 (mean age=17 years 10 months, sd=6 months).

Blood pressure was assessed using a Dinamap 9301 Vital Signs Monitor (Morton Medical, London, UK). Measurements were collected from the right arm while the study participant was seated and at rest. The participant’s arm was supported and an appropriate cuff size was used. Two readings were taken for SBP and DBP. Means were then calculated for use in subsequent analyses.

Metabolic markers were measured in ‘fasting blood’: participants were instructed to fast overnight or for at least 6 h prior to their clinic visit. Blood samples were taken in the clinic following a standardised protocol. Following sample collection, bloods were immediately centrifuged and frozen at −80°C. The samples were assayed 3–9 months later, with no previous freeze–thawing cycles. Metabolic markers were assessed in each sample: plasma lipids were measured using the Lipid Research Clinics Protocol and the Friedewald equation^(^ ^14^ ^)^ was used to calculate LDL-C concentration. Glucose levels were also measured from plasma samples.

### Covariables

Data were collected on a number of other variables that were considered to be potential confounders of any association between diet and CV risk. Those considered included gender, age (at both dietary assessment and outcome measurement), birth weight, gestational age, energy intake at the time of dietary assessment, maternal age at delivery, parity, parental social class and maternal education. Data for gender, birth weight and gestational age at birth were collected by ALSPAC staff at delivery, from medical records or from birth notifications. Maternal data were collected by self-completion postal questionnaires during pregnancy. Maternal education was recorded as the highest completed from Certificate of Secondary Education, vocational training, O-level/General Certificate of Secondary Education (qualifications obtained at 16 years of age), A-levels (qualifications obtained at 18 years of age), degree or higher. Maternal/paternal social class was categorised according to maternal/paternal occupation, respectively. This was done using standardised UK classifications of occupation. Categories were: class I (highest); II; III – non-manual; III – manual; IV; and V (lowest). Exact age (months) of study participants was recorded at clinic visits.

### Ethics

Ethical approval for the study was obtained from the ALSPAC Ethics and Law Committee (ALEC) and the local research ethics committees.

### Statistical analysis

We examined the association of dietary patterns at different time points with CV risk at age 17 years as binary outcomes cross-sectionally by logistic regression modelling.

The International Diabetes Federation advises that adult thresholds for increased CVD risk are applicable to adolescents over the age of 16 years^(^ ^15^ ^,^ ^16^ ^)^. However, threshold values were not available for all of our outcome measures; furthermore, for the majority of measures, few of the cohort members were categorised above these values. We therefore used our own thresholds to indicate increased CV risk within the cohort for consistency and to improve statistical power: we considered those ≥90th centile to be at increased risk. Thresholds used to indicate increased risk were thus ≥133 mmHg for SBP, ≥72 mmHg for DBP, ≥5·51 mmol/l for fasting glucose, ≥28·26 kg/m^2^ for BMI, ≥0·59 mmol/l for LDL-C, ≤0·92 mmol/l for HDL-C, ≥4·67 mmol/l for TC and ≥1·29 mmol/l for TAG.

The first regression model included cluster membership (i.e. dietary pattern) at one time point. Subsequent models adjusted for perinatal (gender, gestational age, birth weight, maternal age at gestation and parity) and sociodemographic variables (maternal education, maternal social class, paternal social class). The final regression models included all covariables plus cluster membership at other ages to assess independent effects of dietary pattern at each age. Odds ratios for the ‘high risk’ group and 95 % confidence intervals for these outcomes were used to evaluate associations between dietary patterns and CV risk measures.

In order to investigate longitudinal associations between cluster membership over time and CV risk outcomes we created a new variable which represented cluster membership over time considering the ‘healthy’ and ‘unhealthy’ diet patterns described at each time point. There were therefore eight different combinations for this variable. Logistic regression was performed as described above using the category of ‘healthy’ at all three time points as the reference group.

All statistical analyses were performed using the statistical software package Stata version 13.

## Results

**Fig. 1:**
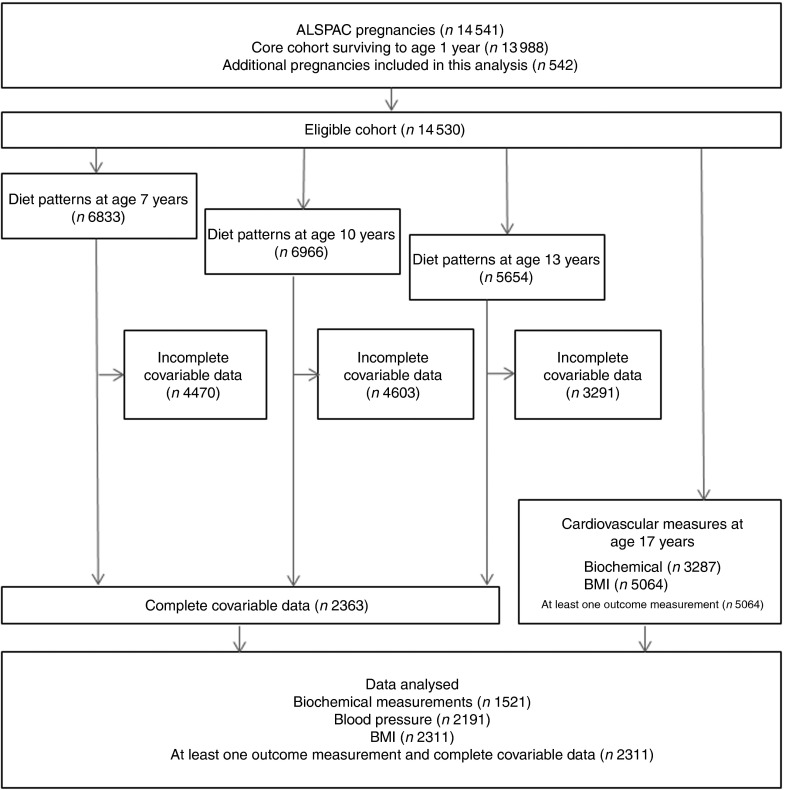
Participant data from the Avon Longitudinal Study of Parents and Children (ALSPAC). Participants were invited to the clinic when they were 7, 10, 13 and 17 years of age. Dietary data were collected when participants were ages 7, 10 and 13 years. Cardiovascular parameters were measured at age 17 years. The present study was conducted using data from participants with at least one cardiovascular parameter measured

From the eligible baseline cohort (*n* 14 530), dietary pattern analysis was conducted on 6833 (47·0 %), 6966 (47·9 %) and 5654 (38·9 %) participants at 7, 10 and 13 years of age respectively. CV parameters were measured on 5064 participants (28·7 %) at age 17 years (mean=17·8 years). The number of participants with CV measures and complete information for dietary patterns at all time points, in addition to covariable data, was 2311 (Fig. 1), of whom 44·1 % were male. Dietary patterns were distributed differently at all time points (age 7, 10 and 13 years) in participants with available outcome data compared with participants with missing data. This was the case for all outcomes measured (*P*<0·001; Table 1).

**Table 1:** Dietary patterns at 7, 10 and 13 years of age in participants in accordance with completeness of cardiovascular risk measurements at 17 years of age; Avon Longitudinal Study of Parents and Children (ALSPAC)

	Outcome data at age 17 years					
	Missing		Complete			
Data type/age/dietary pattern	*n*	%	*n*	%	*χ* ^2^	*P* value
Blood pressure data (SBP/DBP)						
Age 7 years						
‘Healthy’	684	20·38	1024	29·45		
‘Processed’	1096	32·66	894	25·71		
‘Traditional’	728	21·69	830	23·87		
‘Packed lunch’	848	25·27	729	20·97	101·73	6·59×10^−22^
Age 10 years						
‘Healthy’	690	22·11	1287	33·47		
‘Processed’	1067	34·19	1011	26·29		
‘Traditional’	695	22·27	793	20·62		
‘Packed lunch’	669	21·44	754	19·61	119·36	1·06×10^−25^
Age 13 years						
‘Healthy’	519	25·74	1208	33·21		
‘Processed’	730	36·21	1080	29·69		
‘Traditional’	387	19·20	719	19·76		
‘Packed lunch’	380	18·85	621	17·34	42·74	2·79×10^−9^
BMI data						
Age 7 years						
‘Healthy’	619	19·85	1089	29·31		
‘Processed’	1023	32·81	967	26·03		
‘Traditional’	683	21·91	875	23·55		
‘Packed lunch’	793	25·43	784	21·10	103·25	3·11×10^−22^
Age 10 years						
‘Healthy’	623	21·68	1354	33·09		
‘Processed’	998	34·73	1080	26·39		
‘Traditional’	640	22·27	848	20·72		
‘Packed lunch’	613	21·33	810	19·79	120·59	5·75×10^−26^
Age 13 years						
‘Healthy’	445	24·74	1283	33·26		
‘Processed’	659	36·63	1151	29·86		
‘Traditional’	345	19·18	761	19·74		
‘Packed lunch’	350	19·46	661	17·15	50·59	6·00×10^−11^
Fasting blood data (LDL-C/HDL-C/TC/TAG/glucose)						
Age 7 years						
‘Healthy’	959	21·84	749	30·68		
‘Processed’	1375	31·31	615	25·19		
‘Traditional’	991	22·56	567	23·23		
‘Packed lunch’	1067	24·29	510	20·89	77·45	1·08×10^−16^
Age 10 years						
‘Healthy’	1049	24·52	928	34·54		
‘Processed’	1410	32·95	668	24·86		
‘Traditional’	940	21·97	548	20·39		
‘Packed lunch’	880	20·57	543	20·21	96·65	8·17×10^−21^
Age 13 years						
‘Healthy’	831	26·57	896	35·47		
‘Processed’	1076	34·40	734	29·06		
‘Traditional’	624	19·95	482	19·08		
‘Packed lunch’	597	19·09	414	16·39	54·95	7·04×10^−12^

SBP, systolic blood pressure; DBP, diastolic blood pressure; LDL-C, LDL cholesterol; HDL-C, HDL cholesterol; TC, total cholesterol.

### Dietary patterns

Four dietary patterns (‘Healthy’, ‘Processed’, ‘Traditional’ and ‘Packed Lunch’) were previously extracted by CA in ALSPAC participants at ages 7, 10 and 13 years^(^ ^11^ ^)^.

The association between dietary patterns at 7, 10 and 13 years of age and CV risk was investigated by logistic regression analysis, using the ‘Healthy’ dietary pattern as a reference group (Tables 2 and 3). Unadjusted associations between dietary patterns and CV risk were evident at age 7 years: the ‘Processed’ dietary pattern was associated with being in the top 10 % for glucose (*P*=0·04), DBP (*P*=0·01) and BMI (*P*=0·0001) compared with the ‘Healthy’ dietary pattern at this time point. The ‘Traditional’ dietary pattern at age 7 years was associated with being in the top 10 % for BMI (*P*=0·05) compared with the ‘Healthy’ dietary pattern. The ‘Packed lunch’ dietary pattern at age 7 years was associated with SBP (*P*=0·01), DBP (*P*=0·03), BMI (*P*<0·0001) and HDL-C (*P*=0·02). At age 10 years, compared with the ‘Healthy’ dietary pattern, the ‘Traditional’ dietary pattern was crudely associated with being in the top 10 % for SBP (*P*=0·03) and inversely associated with being in the top 10 % for TC (*P*=0·003), whereas the ‘Packed lunch’ pattern was associated with increased BMI (*P*=0·004). Finally, for dietary patterns at age 13 years: the ‘Processed’ dietary pattern was associated with being in the top 10 % for DBP (*P*=0·02) and BMI (*P*=0·001); the ‘Traditional’ dietary pattern was associated with higher HDL-C (*P*=0·03); and the ‘Packed lunch’ dietary pattern was positively associated with being in the top 10 % for SBP (*P*=0·0001), DBP (*P*=0·01), BMI (*P*=0·002) and HDL-C (*P*=0·03) and inversely associated with higher levels of TC (*P*=0·04; all compared with the ‘Healthy’ dietary pattern).

**Table 2:** Logistic regression model: diet patterns and physiological outcomes; Avon Longitudinal Study of Parents and Children (ALSPAC). Unadjusted and adjusted OR and 95 % CI and *P* values for being above the 90th centile (value shown in parentheses) for each outcome

	SBP (≥133 mmHg)			DBP (≥72 mmHg)			BMI (≥28·26 kg/m^2^)		
Model/age/dietary pattern	OR	95 % CI	*P* value	OR	95 % CI	*P* value	OR	95 % CI	*P* value
Model 1									
Age 7 years									
‘Healthy’	Reference			Reference			Reference		
‘Processed’	1·01	0·74, 1·36	0·97	1·51	1·12, 2·05	0·01	1·89	1·36, 2·62	0·0001
‘Traditional’	1·05	0·77, 1·43	0·76	1·21	0·87, 1·67	0·25	1·41	1·00, 2·01	0·05
‘Packed lunch’	1·49	1·10, 2·00	0·01	1·44	1·04, 1·98	0·03	2·40	1·73, 3·32	1·67×10^−7^
Age 10 years									
‘Healthy’	Reference			Reference			Reference		
‘Processed’	1·05	0·79, 1·38	0·74	0·98	0·75, 1·28	0·88	1·13	0·85, 1·50	0·41
‘Traditional’	1·38	1·04, 1·82	0·03	0·82	0·61, 1·10	0·19	1·03	0·75, 1·41	0·86
‘Packed lunch’	1·13	0·84, 1·53	0·41	0·93	0·70, 1·25	0·64	1·54	1·15, 2·05	0·004
Age 13 years									
‘Healthy’	Reference			Reference			Reference		
‘Processed’	1·11	0·86, 1·53	0·37	1·40	1·07, 1·85	0·02	1·63	1·23, 2·16	0·001
‘Traditional’	1·27	0·93, 1·74	0·14	0·75	0·52, 1·07	0·12	1·21	0·87, 1·68	0·26
‘Packed lunch’	1·84	1·36, 2·50	0·0001	1·56	1·14, 2·12	0·01	1·66	1·20, 2·28	0·002
Model 2*									
Age 7 years									
‘Healthy’	Reference			Reference			Reference		
‘Processed’	0·80	0·52, 1·24	0·32	1·15	0·75, 1·77	0·52	1·60	1·01, 2·55	0·05
‘Traditional’	0·91	0·60, 1·38	0·66	1·25	0·82, 1·91	0·29	1·22	0·75, 1·98	0·42
‘Packed lunch’	1·32	0·86, 2·02	0·21	1·44	0·92, 2·26	0·11	1·96	1·22, 3·13	0·005
Age 10 years									
‘Healthy’	Reference			Reference			Reference		
‘Processed’	1·17	0·77, 1·78	0·47	0·87	0·59, 1·29	0·48	0·80	0·52, 1·23	0·31
‘Traditional’	1·44	0·96, 2·15	0·08	0·64	0·42, 0·99	0·05	0·82	0·52, 1·27	0·37
‘Packed lunch’	0·89	0·56, 1·42	0·63	0·70	0·44, 1·09	0·12	0·97	0·62, 1·52	0·89
Age 13 years									
Healthy	Reference			Reference			Reference		
‘Healthy’	0·98	0·65, 1·46	0·91	1·19	0·81, 1·74	0·38	1·15	0·77, 1·72	0·51
‘Processed’	0·87	0·57, 1·33	0·52	0·80	0·50, 1·26	0·33	1·00	0·63, 1·59	1·00
‘Traditional’	1·17	0·75, 1·83	0·49	1·53	0·98, 2·40	0·06	1·41	0·87, 2·28	0·16

SBP, systolic blood pressure; DBP, diastolic blood pressure.

* Model 2 adjusts for: age at time of dietary assessment, age at time of outcome measurement, gender, birth weight, gestational age, energy intake at time of dietary assessment, maternal age and parity, mother’s social class, father’s social class, maternal education and dietary patterns at other ages.

**Table 3:** Logistic regression model: diet patterns and biochemical outcomes; Avon Longitudinal Study of Parents and Children (ALSPAC). Unadjusted and adjusted OR and 95 % CI and *P* values for being above the 90th centile (value shown in parentheses) for each outcome

	LDL-C (≥0·59 mmol/l)			HDL-C (≤0·92 mmol/l)			TAG (≥1·29 mmol/l)			TC (≥4·67 mmol/l)			Glucose (≥5·51 mmol/l)		
Model/age/dietary pattern	OR	95 % CI	*P* value	OR	95 % CI	*P* value	OR	95 % CI	*P* value	OR	95 % CI	*P* value	OR	95 % CI	*P* value
Model 1															
Age 7 years															
Healthy	Reference			Reference			Reference			Reference			Reference		
‘Healthy’	0·80	0·55, 1·16	0·24	1·08	0·74, 1·56	0·70	0·81	0·56, 1·15	0·25	0·99	0·69, 1·41	0·95	1·48	1·03, 2·12	0·04
‘Processed’	0·88	0·60, 1·27	0·48	1·18	0·81, 1·71	0·40	0·84	0·58, 1·23	0·37	1·00	0·70, 1·44	0·98	1·11	0·75, 1·64	0·60
‘Traditional’	0·96	0·66, 1·40	0·84	1·54	1·07, 2·21	0·02	0·93	0·64, 1·35	0·70	0·93	0·64, 1·37	0·72	1·03	0·68, 1·56	0·89
Age 10 years															
Healthy	Reference			Reference			Reference			Reference			Reference		
‘Healthy’	0·80	0·57, 1·13	0·20	1·09	0·78, 1·52	0·61	0·80	0·57, 1·13	0·21	0·97	0·71, 1·32	0·82	0·88	0·63, 1·24	0·47
‘Processed’	0·80	0·56, 1·16	0·24	1·00	0·70, 1·43	0·99	0·76	0·52, 1·10	0·14	0·56	0·38, 0·82	0·003	1·03	0·73, 1·47	0·85
‘Traditional’	1·04	0·74, 1·47	0·80	1·24	0·89, 1·75	0·21	1·03	0·73, 1·45	0·86	0·78	0·55, 1·11	0·16	1·00	0·70, 1·43	0·99
Age 13 years															
Healthy	Reference			Reference			Reference			Reference			Reference		
‘Healthy’	0·85	0·61, 1·19	0·35	1·40	1·01, 1·94	0·05	0·89	0·64, 1·25	0·51	0·76	0·55, 1·04	0·09	1·04	0·75, 1·45	0·81
‘Processed’	1·05	0·73, 1·51	0·80	1·50	1·04, 2·15	0·03	1·04	0·72, 1·50	0·84	0·92	0·65, 1·30	0·64	1·02	0·70, 1·48	0·93
‘Traditional’	1·05	0·72, 1·54	0·80	1·37	0·93, 2·02	0·11	1·09	0·75, 1·60	0·65	0·65	0·44, 0·98	0·04	1·09	0·74, 1·06	0·66
Model 2*															
Age 7 years															
Healthy	Reference			Reference			Reference			Reference			Reference		
‘Healthy’	0·54	0·32, 0·90	0·02	1·25	0·74, 2·10	0·40	0·53	0·32, 0·89	0·02	1·01	0·62, 1·65	0·96	1·61	0·96, 2·69	0·07
‘Processed’	0·83	0·53, 1·32	0·44	1·41	0·86, 2·32	0·17	0·82	0·52, 1·30	0·41	0·98	0·61, 1·58	0·94	1·20	0·71, 2·04	0·50
‘Traditional’	0·64	0·37, 1·09	0·10	1·83	1·09, 3·05	0·02	0·60	0·35, 1·04	0·07	1·02	0·60, 1·73	0·94	1·17	0·66, 2·08	0·59
Age 10 years															
Healthy	Reference			Reference			Reference			Reference			Reference		
‘Healthy’	0·53	0·32, 0·87	0·01	0·92	0·57, 1·49	0·74	0·52	0·32, 0·87	0·01	0·76	0·48, 1·20	0·24	0·47	0·27, 0·83	0·01
‘Processed’	0·62	0·38, 1·01	0·06	0·67	0·40, 1·11	0·12	0·58	0·35, 0·95	0·03	0·50	0·30, 0·84	0·01	0·74	0·45, 1·22	0·24
‘Traditional’	0·63	0·37, 1·05	0·08	0·93	0·56, 1·54	0·77	0·66	0·40, 1·11	0·12	0·41	0·23, 0·73	0·002	0·67	0·39, 1·17	0·16
Age 13 years															
Healthy	Reference			Reference			Reference			Reference			Reference		
‘Healthy’	0·67	0·41, 1·07	0·10	1·17	0·73, 1·86	0·52	0·70	0·44, 1·13	0·15	0·73	0·46, 1·17	0·19	0·71	0·44, 1·16	0·17
‘Processed’	0·93	0·57, 1·50	0·75	1·32	0·82, 2·14	0·25	0·91	0·56, 1·48	0·70	1·06	0·67, 1·70	0·80	0·84	0·50, 1·40	0·50
‘Traditional’	1·05	0·62, 1·80	0·85	1·02	0·58, 1·78	0·96	1·08	0·63, 1·86	0·77	1·02	0·58, 1·80	0·94	0·70	0·39, 1·26	0·23

LDL-C, LDL cholesterol; HDL-C, HDL cholesterol; TC, total cholesterol.

* Model 2 adjusts for: age at time of dietary assessment, age at time of outcome measurement, gender, birth weight, gestational age, energy intake at time of dietary assessment, maternal age and parity, mother’s social class, father’s social class, maternal education and dietary patterns at other ages

After adjustment for potential confounders, all associations were attenuated except for the association between the ‘Processed’ and ‘Packed lunch’ clusters at age 7 years and being in the top 10 % for BMI, compared with individuals in the ‘Healthy’ diet pattern group (OR=1·60, *P*=0·05 and OR 1·96, *P*=0·005, respectively; Table 2).

For the longitudinal analysis we chose to combine the ‘Healthy’ and ‘Traditional’ patterns together and the ‘Processed’ and ‘Packed lunch’ patterns together to create either ‘healthy’ or ‘unhealthy’ patterns at each age. Table 4 shows the association between the longitudinal variable created using ‘healthy’/’unhealthy’ at age 7, 10 and 13 years and BMI at age 17 years. No other associations were evident with this variable and CV risk factors (data not shown). Compared with those children who were in a ‘healthy’ cluster at all three time points, children who were in an ‘unhealthy’ cluster at age 7 years were more likely to be in the top decile of BMI at age 17 years, regardless of their later cluster membership.

**Table 4:** Logistic regression model: stability of diet patterns over time (combination of ‘healthy’ or ‘unhealthy’ pattern at the three ages of interest) and BMI; Avon Longitudinal Study of Parents and Children (ALSPAC). Unadjusted and adjusted OR and 95 % CI and *P* values

Diet pattern combination	Model 1				Model 2*			
(age 7, 10 and 13 years)	*n*	OR	95 % CI	*P* value	*n*	OR	95 % CI	*P* value
H, H, H	939	Reference			631	Reference		
H, H, U	390	1·51	0·88, 2·61	0·135	244	1·46	0·78, 2·75	0·241
H, U, H	315	1·38	0·74, 2·56	0·309	200	1·09	0·52, 2·25	0·823
H, U, U	362	1·72	0·99, 3·00	0·056	211	1·88	1·00, 3·53	0·049
U, H, H	384	2·14	1·28, 3·57	0·004	234	1·98	1·10, 3·58	0·023
U, H, U	399	2·78	1·70, 4·53	<0·0001	212	2·79	1·57, 5·00	0·001
U, U, H	428	2·25	1·37, 3·71	0·001	253	2·06	1·14, 3·71	0·016
U, U, U	756	2·37	1·53, 3·67	<0·0001	378	1·65	0·94, 2·88	0·080

H, ‘healthy’ (either ‘Healthy’ or ‘Traditional’ dietary pattern); U, ‘unhealthy’ (either ‘Processed’ or ‘Packed lunch’ dietary pattern).

* Model 2 adjusts for: age at time of dietary assessment, age at time of outcome measurement, gender, birth weight, gestational age, energy intake at time of dietary assessment, maternal age and parity, mother’s social class, father’s social class and maternal education.

## Discussion

Our results suggest that dietary patterns, measured at ages 7, 10 and 13 years using CA, have little overall contribution to measures of CV risk assessed at age 17 years. The only association we found was between dietary pattern membership at age 7 and BMI at age 17. Results from regression analyses were consistent regardless of whether outcomes were investigated as continuous or binary measures. Although the importance of diet as a risk factor for CVD has not been illustrated here, there is some evidence to suggest that CV risk is modifiable by diet and in particular that childhood nutrition may influence the progression of CVD later in life^(^ ^17^ ^,^ ^18^ ^)^. In one study it was found that dietary patterns were associated with CV risk factors and vascular markers for subclinical atherosclerosis^(^ ^17^ ^)^. Similarly, Mikkila *et al*. found that a dietary pattern associated with ‘health conscious’ food choices was inversely associated with CV risk in the Young Finns Study^(^ ^18^ ^)^. However, outcome variables were investigated in adults and not adolescents in these studies. In addition, since both studies were conducted using data from the Young Finns Study, the findings may not be transferable to our cohort.

The influence of diet on disease risk is difficult to extract. It has previously been reported that socio-economic status is linked to vascular disease^(^ ^3^ ^,^ ^19^ ^,^ ^20^ ^)^ and dietary patterns in ALSPAC have been found to correlate with a number of socio-economic factors^(^ ^21^ ^,^ ^22^ ^)^. In addition, a recent review concluded that obesogenic behaviours (such as diet behaviours and sociodemographic indicators) cluster in children and adolescents^(^ ^23^ ^)^. We therefore took measures to limit confounding by socio-economic status by adjusting for parental education and social class in our final analysis. However, we cannot rule out the possibility that residual confounding may affect the interpretation of our results.

When analysing a sub-population within a cohort it is important to consider how these participants may differ from the rest of the study cohort. As dietary patterns are distributed differently in participants with available outcome data compared with those without, it is likely that the present study is affected by loss to follow-up bias: it is possible that children with the less healthy dietary patterns are under-represented in our cohort.

As CV events in children are extremely rare, intermediate measures of CVD may be assessed to make inferences regarding vascular health^(^ ^24^ ^)^. Due to the youth of the cohort, it may be that our study is underpowered to detect an association between dietary patterns and CV measures. There is little variation in the CV risk factors (with the exception of BMI) in this cohort at this age. The methodology outlined here will therefore be repeated on data that will be collected when the cohort is aged 24–25 years in order to investigate the relationship between diet and vascular health as the cohort ages and the variation in the measures increases. Nevertheless, we did find an association between dietary pattern at age 7 years and subsequent BMI at age 17 years, such that children in the ‘Processed’ and ‘Packed lunch’ clusters had higher BMI on average and were at increased risk of being in the top decile. It is possible that this was a chance finding, given the number of outcomes we investigated. However, when looking longitudinally it became evident that being in an ‘unhealthy’ pattern (either ‘Processed’ or ‘Packed lunch’) at age 7 years was associated with being in the top decile of BMI at age 17 years regardless of what pattern they were in at later ages. This suggests that 7 years of age may be a critical period in the life course for the development of obesity in early adulthood.

Dietary patterns (from FFQ) have been used successfully in ALSPAC to extrapolate the relationship between diet at 6, 15 and 24 months of age and intelligence quotient at 8 years of age^(^ ^25^ ^)^. Similarly, it could be that diet earlier in life (rather than at 7, 10 or 13 years of age) has a greater effect on CV phenotypes and therefore may explain why we did not observe any associations in our analysis as we limited our exposure variables to the three time points where dietary data were collected on the whole cohort using diet diaries only.

Longitudinal dietary data are necessary to establish any critical time point(s) at which dietary intake is most important for CV health and to establish whether adhering to one type of dietary pattern over a period of time, or changing to a different dietary pattern, renders an individual more or less likely to be at risk of disease. It has been reported previously that dietary patterns track throughout early and mid-childhood in ALSPAC, the most stable pattern being the ‘Healthy’ pattern followed by the ‘Processed’ pattern^(^ ^8^ ^,^ ^11^ ^)^. As dietary data for ALSPAC participants in adolescence are unavailable we are unable to remark on how dietary patterns track from childhood into adolescence. However, in a Chinese population it was concluded that children are likely to maintain dietary patterns from childhood to adolescence, regardless of socio-economic change^(^ ^7^ ^)^. We would like to extend this analysis to investigate how cluster stability influences CV risk. Tracking over time is easier to quantify for patterns that have been derived using CA as this method groups individuals to one category only at each time point. A change in category can therefore be easily determined. Further research is underway to model these data longitudinally and we will follow up in the cohort.

Diet is a complex exposure variable; in an attempt to capture the influence of diet as a whole we used dietary patterns, derived by CA, to investigate associations between diet and CV risk measures. In summary, we did not find evidence to support an association between dietary patterns in childhood and CV measures in adolescence, with the exception of BMI. However, differences may become clearer in later life and we would encourage the adoption of a healthy diet as early in life as possible.
